# Identification of circular RNAs as a promising new class of diagnostic biomarkers for human breast cancer

**DOI:** 10.18632/oncotarget.17307

**Published:** 2017-04-21

**Authors:** Lingshuang Lü, Jian Sun, Peiyi Shi, Weimin Kong, Kun Xu, Biyu He, Simin Zhang, Jianming Wang

**Affiliations:** ^1^ Department of Epidemiology, School of Public Health, Nanjing Medical University, Nanjing, 211166, China; ^2^ Department of Thoracic Surgery, The First People′s Hospital of Yancheng City, Yancheng, 224001, China; ^3^ Department of Social Medicine and Health Education, School of Public Health, Nanjing Medical University, Nanjing, 211166, China

**Keywords:** breast cancer, RNA, noncoding RNA, circRNA, biomarker

## Abstract

Endogenous noncoding circular RNAs (circRNAs) have gained attention for their involvement in carcinogenesis, but their expression pattern in breast cancer has remained largely unknown. In this two-stage study, we first used an Arraystar Human circRNA Array to construct a genome-wide circRNA profile. We then selected candidate circRNAs for validation using a quantitative real-time polymerase chain reaction system. CircRNA/miRNA interactions were predicted and sequence analyses were performed. Among 1155 differentially expressed circRNAs, 715 were upregulated and 440 were downregulated in breast cancer tissues. The validation study demonstrated that hsa_circ_103110, hsa_circ_104689 and hsa_circ_104821 levels were elevated in breast cancer tissues, whereas hsa_circ_006054, hsa_circ_100219 and hsa_circ_406697 were downregulated. These circRNAs targeted complementary miRNA response elements. The area under the receiver operating characteristic curve for distinguishing breast cancer was 0.82 (95% CI: 0.73-0.90) when hsa_circ_006054, hsa_circ_100219 and hsa_circ_406697 were used in combination. This study provides evidence that circRNAs are differentially expressed in breast cancer and are important in carcinogenesis because they participate in cancer-related pathways and sequester miRNAs.

## INTRODUCTION

Breast cancer is the most frequently occurring cancer and the leading cause of cancer-related death among women worldwide, with an estimated 1.7 million incident cases and 521,900 deaths in 2012 [1]. Epidemiological studies have shown that obesity, advanced maternal age at first birth, estrogen and progestin use, physical inactivity and alcohol consumption are associated with an increased risk of breast cancer in women [2–5]. Some of these factors also influence the prognosis of patients after treatment. However, people sharing the same conditions and familial aggregation have different lifetime risks, indicating that genetic factors are essential to breast cancer etiology [6, 7].

Besides genetic mutations, epigenetic mechanisms are also important in the tumorigenesis of breast cancer [8–10]. Epigenetic modifications include DNA methylation, histone modification and noncoding RNA (ncRNA). A significant fraction of the human genome is transcribed as ncRNAs [11]. The functional relevance of ncRNAs is particularly evident for microRNAs (miRNAs) and long noncoding RNAs (lncRNAs) [12]. Over the last few years, reports have indicated that miRNAs are involved in all aspects of the carcinogenic process and can be used as biomarkers for early risk stratification and long-term survival prediction [13, 14]. Specific miRNA expression profiles can enable accurate tumor classification, targeted therapy and individualized intervention [15, 16].

Circular RNA (circRNA) is another novel class of endogenous ncRNA molecules [17]. CircRNAs were found in viroids, viruses and tetrahymena decades ago, but they were initially considered to be by-products of aberrant RNA splicing or splicing errors due to their low expression [18, 19]. Thanks to technological breakthroughs in high-throughput sequencing and computational approaches, circRNAs have drawn increasing interest, especially with the discovery of their vast abundance, diverse functions, and frequent tissue-specific expression [20, 21]. The expression of circRNAs is outstandingly high compared with the expression of linear RNA isoforms of the same genes [22, 23]. Recent evidence has indicated that circRNAs can regulate gene expression by sequestering specific miRNAs or buffering their repression of mRNA targets [17, 24]. For example, the circRNA Cdr1as, also known as CiRS-7, has been shown to act as a powerful miR-7 sponge/inhibitor in the developing midbrain of zebrafish [25].

Aberrant expression of circRNAs has been shown to occur in colorectal, basal cell and bladder carcinoma [26–28]. Recently, the involvement of circRNAs in breast cancer has also been explored, but only preliminary findings have been reported and there has been a lack of experimental and clinical evidence [29, 30]. Nair et al. developed a Circ-Seq workflow to identify expressed circRNAs, and found that circRNAs may be markers of cell proliferation in breast cancer and associate with cancer subtypes [30]. Galasso et al. used a bioinformatics detection tool to explore the predictive value of circRNAs in breast cancer, but the size of the cohort was small [29].

Here, we performed a molecular epidemiological study in a Chinese population to establish the circRNA expression profile and identify deregulated circRNAs in the carcinogenesis of human breast cancer.

## RESULTS

### Characteristics of the study population

In the first stage, we used the Arraystar Human circRNA Array to sequence four paired breast cancer samples from patients with invasive ductal breast cancer (Supplementary Table 1). In the validation stage, we recruited 51 breast cancer patients. The mean (range) age was 46.5 (18-79) years. The characteristics of the patients are presented in Table 1.

**Table 1:** Characteristics of study subjects in the validation study (n=51)

Characteristics	Number	%
Age (years)		
≤60	31	60.78
>60	20	39.22
ER		
positive	26	50.98
negative	25	49.02
PR		
positive	19	37.25
negative	32	62.75
HER2		
positive	30	58.82
negative	21	41.18
Tumor stage		
T1	16	31.37
T2	30	58.82
T3	5	9.80
Lymphatic metastasis		
N0	25	49.02
N1	17	33.33
N2	9	17.65

ER: estrogen receptor; PR: progestin receptor; HER2: human epidermal growth factor receptor-2.

### Findings from the circRNA microarray

**Figure 1:**
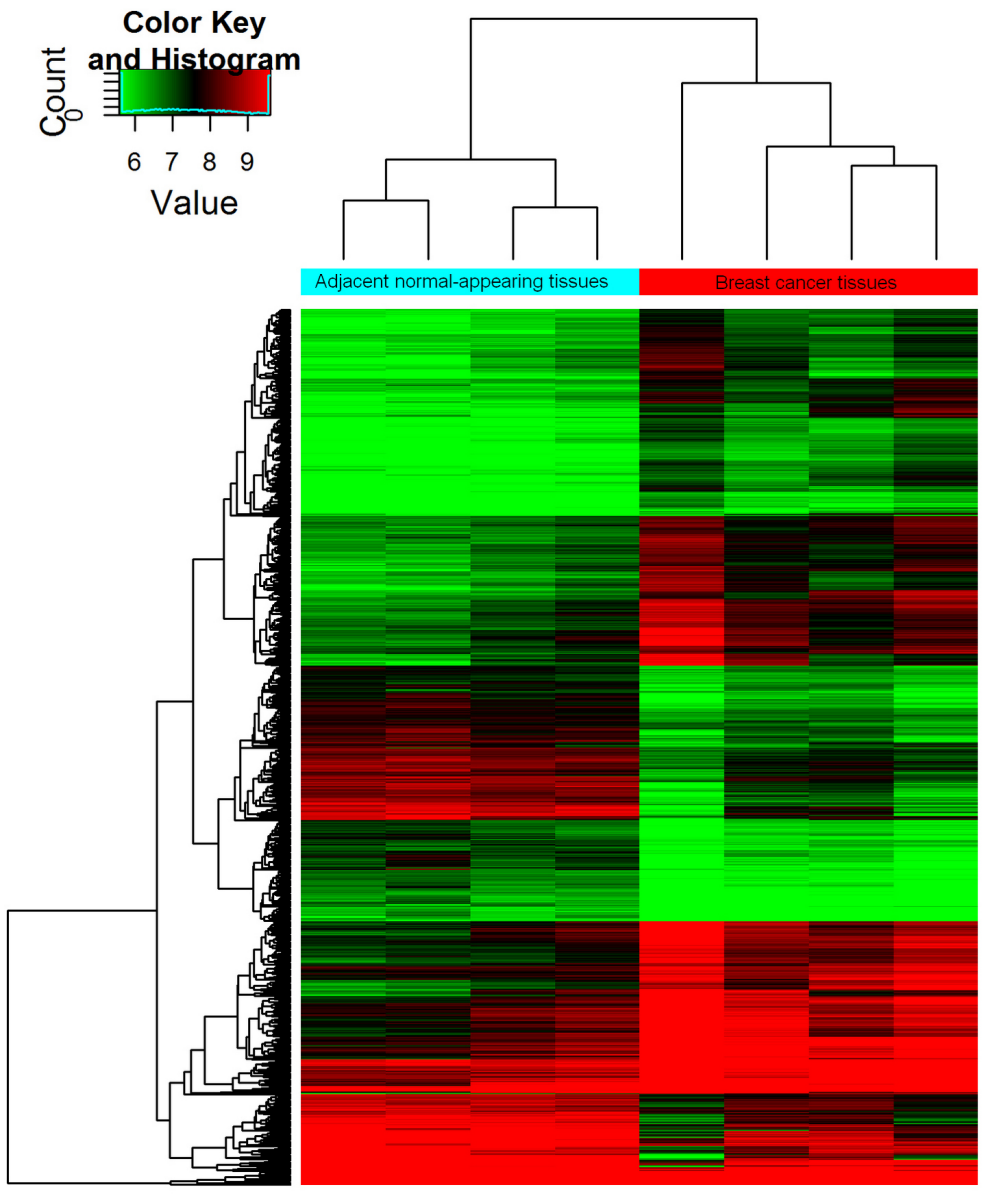
Hierarchical cluster of circRNA expression profiles from the microarray The color scale runs from green (low intensity), through black (medium intensity), to red (strong intensity).

**Figure 2:**
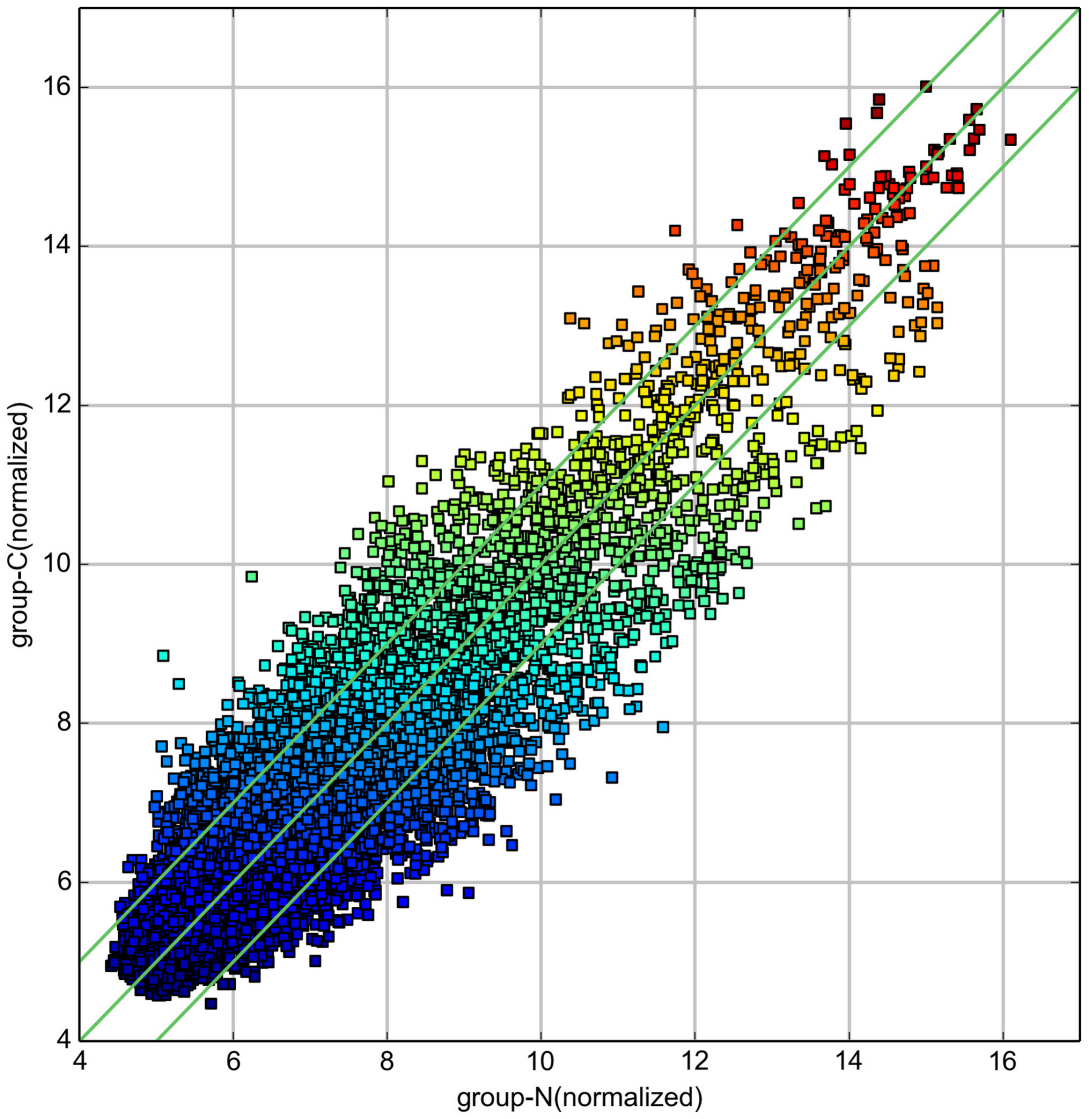
Scatterplot of circRNA signal values between breast cancer and adjacent normal-appearing tissues The values spotted in the X and Y axes represent the normalized signals of samples in the two groups (log2-scaled). The green lines represent fold-changes. The circRNAs above the upper green line and below the lower green line are those with expression fold-changes >2.0 between breast cancer and normal-appearing tissues.

**Figure 3:**
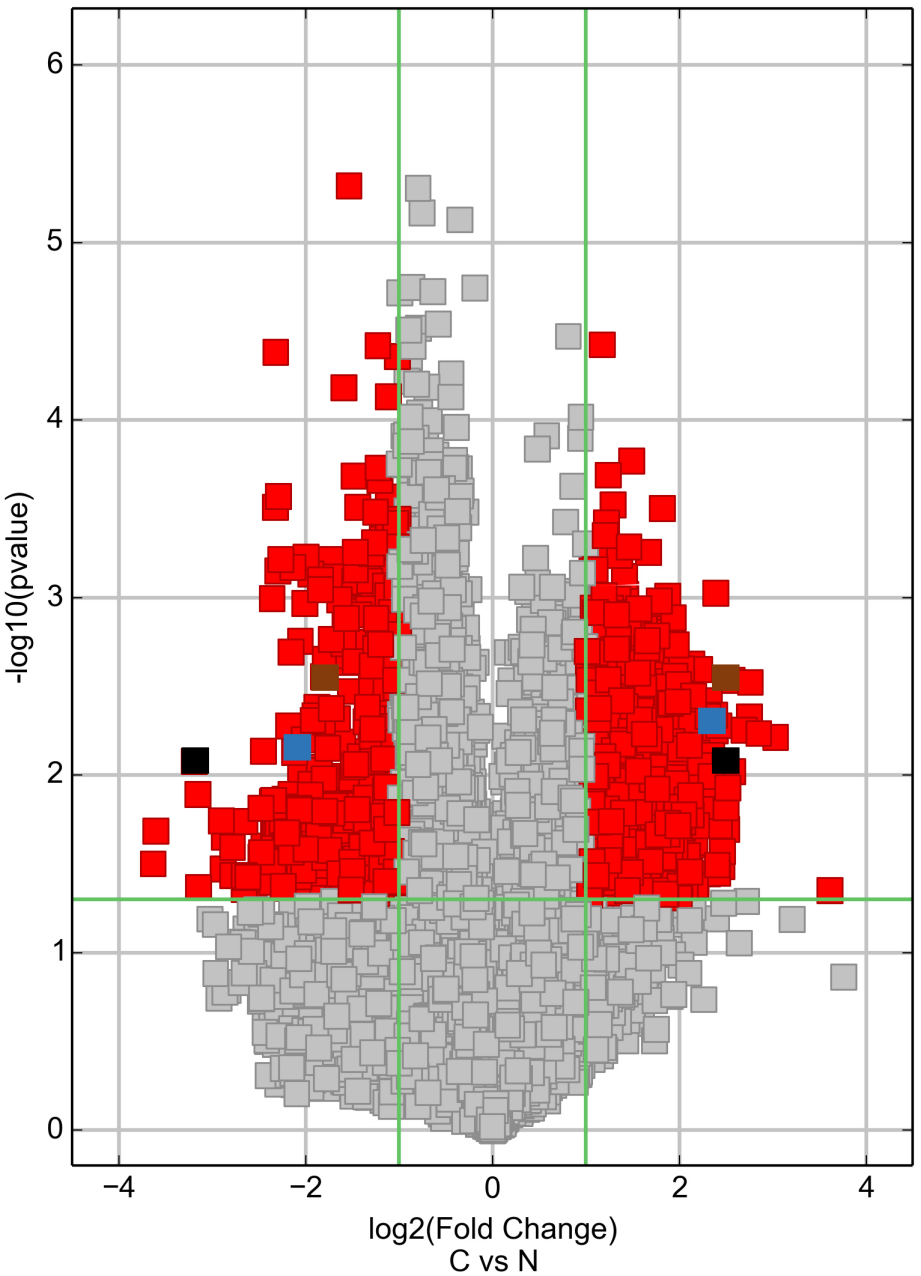
Volcano plot visualizing the differential expression of circRNAs between breast cancer and adjacent normal-appearing tissues The vertical green lines correspond to up- and downregulation >2.0-fold. The horizontal green line represents a P-value of 0.05. The red squares indicate differentially expressed circRNAs with statistical significance. On the right side, the brown square represents hsa_circ_103110, the blue square represents hsa_circ_104689, and the black square represents hsa_circ_104821. On the left side, the brown square represents hsa_circ_100219, the blue square represents hsa_circ_006054, and the black square represents hsa_circ_406697. C: breast cancer tissue. N: adjacent normal-appearing tissue.

Differentially expressed circRNAs were detected in the four matched tissue samples. When we set the filter criteria as a fold-change ≥2 and a P-value <0.05, we found that a total of 715 circRNAs were significantly upregulated and 440 circRNAs were significantly downregulated in the breast cancer lesions compared with adjacent normal-appearing tissues. Considering that false positives can be caused by multi-comparisons, we used the false discovery rate (FDR) method to adjust the P values. After FDR correction, a total of 16 circRNAs were found to be significantly upregulated, while 5 circRNAs were significantly downregulated. Figure 1 is a hierarchical cluster displaying the levels of circRNAs in breast cancer and adjacent normal-appearing tissues. Figure 2 is a scatterplot describing the variation in circRNA expression between the two groups. Figure 3 is a volcano plot visualizing the statistical significance of differentially expressed circRNAs between cases and controls.

### Validation of deregulated circRNAs

**Figure 4:**
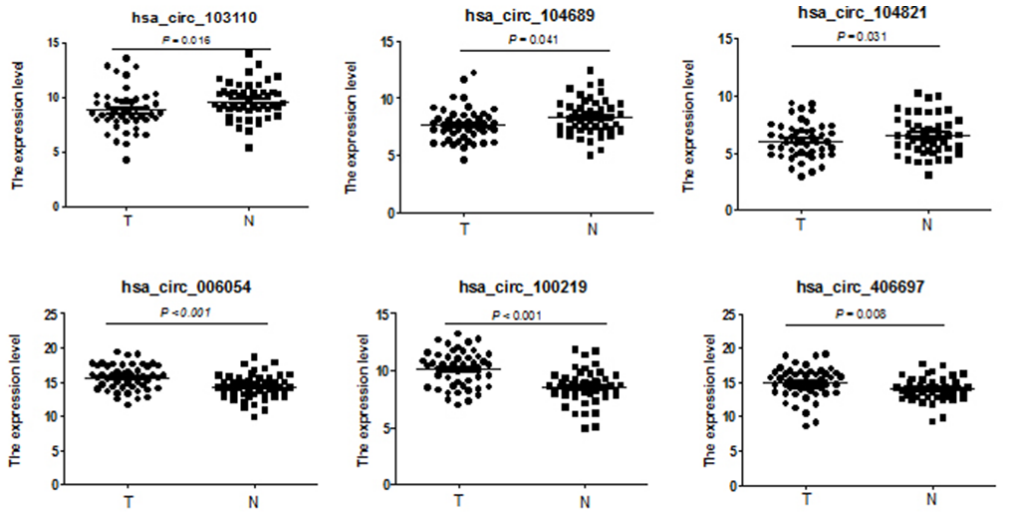
Validation of novel circRNAs by qRT-PCR in breast cancer and adjacent normal-appearing tissues Scatterplots display the relative expression of specific circRNAs between breast cancer (T) and adjacent normal-appearing tissues (N). Marks above the reference line indicate downregulated circRNAs.

**Figure 5:**
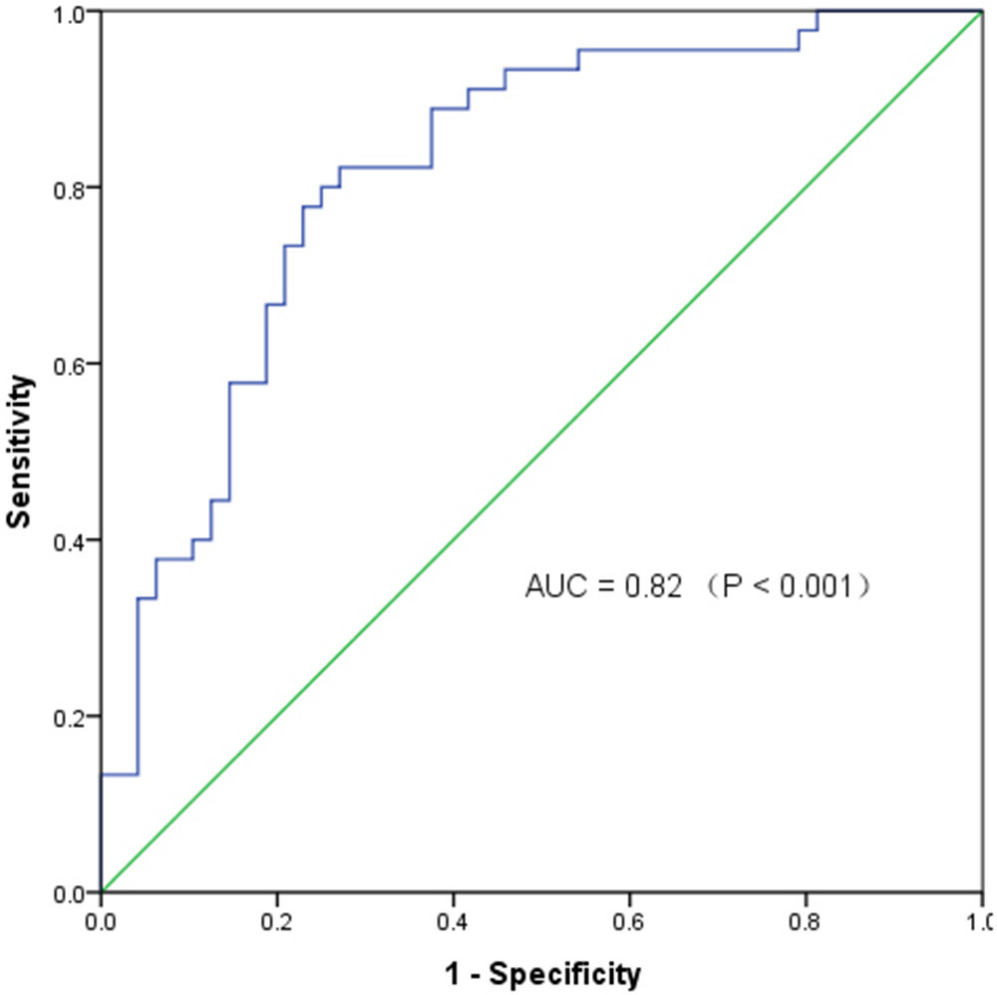
The receiver operating characteristic curve of specific circRNAs in distinguishing breast cancer AUC: area under the curve; Combination of circRNAs: hsa_circ_006054, hsa_circ_100219 and hsa_circ_406697.

To test whether the differentially expressed circRNAs discovered through the microarray were bona fide, we selected six potentially significant circRNAs for validation by the quantitative real-time reverse transcription PCR (qRT-PCR). We used the following criteria: (1) length around 200 to 3000 bp; (2) fold-change >2; (3) p-value <0.01; (4) raw intensity >200; (5) exonic-related circRNAs; and (6) conservative. These six circRNAs are highlighted in the volcano plot (Figure 3). The scatterplot revealed that hsa_circ_103110, hsa_circ_104689 and hsa_circ_104821 levels were elevated in cancer tissues, whereas hsa_circ_006054, hsa_circ_100219 and hsa_circ_406697 were downregulated in breast cancer lesions (Figure 4). Subgroup analysis demonstrated that progestin receptor negativity (PR-) was associated with upregulation of hsa_circ_104689 and hsa_circ_104821, and with downregulation of hsa_circ_406697 (Supplementary Table 2). We further evaluated the diagnostic value of these six circRNAs for breast cancer by plotting a receiver operating characteristic curve. We found that hsa_circ_100219 had the highest diagnostic accuracy, with an area under the curve (AUC) of 0.78 (95% CI: 0.69-0.88) (Table 2). When hsa_circ_006054, hsa_circ_100219 and hsa_circ_406697 were combined, the AUC increased to 0.82 (95% CI: 0.73-0.90) (Figure 5).

**Table 2:** Expression and diagnostic value of specific circRNAs in breast cancer

CircRNAs	Expression^a^		t	*P*	AUC (95% CI)	Sensitivity (95% CI)	Specificity (95% CI)	Cut-off value^b^
	Breast cancer	Adjacent tissues						
hsa_circ_103110	8.79±1.86	9.62±1.63	2.49	0.016	0.63 (0.52–0.74)	0.63 (0.48–0.76)	0.63 (0.48–0.76)	8.97
hsa_circ_104689	7.66±1.48	8.31±1.57	2.09	0.041	0.61 (0.50–0.73)	0.57 (0.42–0.71)	0.55 (0.40–0.69)	7.67
hsa_circ_104821	6.07±1.55	6.52±1.68	2.22	0.031	0.60 (0.49–0.71)	0.57 (0.42–0.71)	0.57 (0.42–0.71)	6.04
hsa_circ_006054	15.66±1.82	14.29±1.75	4.30	<0.001^c^	0.71 (0.61–0.81)	0.65 (0.50–0.78)	0.69 (0.54–0.81)	14.84
hsa_circ_100219	10.13±1.58	8.53±1.43	5.70	<0.001^c^	0.78 (0.69–0.88)	0.69 (0.54–0.81)	0.71 (0.56–0.83)	8.95
hsa_circ_406697	14.85±2.27	13.96±1.68	2.76	0.008^c^	0.64 (0.52–0.75)	0.63 (0.48–0.76)	0.63 (0.48–0.76)	14.24

^a^: The expression of each circRNA was calculated from the ΔCt and expressed as mean±standard deviation; ^b^: We used the median as the cut-off value; ^c^: Significant after Bonferroni correction (P<0.008); AUC: area under the curve; CI: confidence interval.

### GO enrichment and KEGG analysis

To explore how circRNAs regulate parental gene transcription, we performed Gene Ontology (GO) enrichment analysis for the genes targeted by the circRNAs that were found to be differentially expressed in our qRT-PCR results. Target genes of upregulated circRNAs in breast cancer were involved in the developmental process, positive regulation of gene expression and positive regulation of biological processes (Supplementary Figure 1A), whereas target genes of downregulated circRNAs in breast cancer were involved in the transmembrane receptor protein tyrosine kinase signaling pathway and the developmental process (Supplementary Figure 1B). Kyoto Encyclopedia of Genes and Genomes (KEGG) analysis revealed that pathways such as the Hippo signaling pathway and the WNT signaling pathway were related to the upregulated circRNAs, while pathways such as the RAP1 signaling pathway and the RAS signaling pathway were related to the downregulated circRNAs (Supplementary Figure 2A & 2B).

### MiRNA response element (MRE) sequence analysis

As shown in Table 3, dominant MREs were targeted by differentially expressed circRNAs. Some of these miRNAs have been reported to be associated with breast cancer [31–38]. By referring to published literature, we identified the following miRNA-circRNA matches: hsa_miR_339_5p and hsa_circ_103110; hsa_miR_143_5p and hsa_circ_104689; hsa_miR_409_3p/hsa_miR_153_3p/hsa_miR_145_5p and hsa_circ_104821; hsa_miR_298/hsa_miR_485_3p and hsa_circ_100219; and hsa_miR_100_3p and hsa_circ_406697. MRE sequence analysis for the differentially expressed circRNAs revealed a variety of features boosting MRE efficacy (Supplementary Figure 3).

**Table 3:** The differentially expressed circRNAs and microRNA response elements

CircRNAs	Alias (circBase)	MRE1	MRE2	MRE3	MRE4	MRE5
hsa_circ_103110	hsa_circ_0004771	hsa-miR-653–5p	hsa-miR-339–5p	hsa-miR-330–5p	hsa-miR-595	hsa-miR-629–3p
hsa_circ_104689	hsa_circ_0001824	hsa-miR-627–3p	hsa-miR-143–5p	hsa-miR-656–3p	hsa-miR-376a-5p	hsa-miR-222–5p
hsa_circ_104821	hsa_circ_0001875	hsa-miR-409–3p	hsa-miR-153–3p	hsa-miR-145–5p	hsa-miR-9–5p	hsa-miR-152–3p
hsa_circ_006054	hsa_circ_0006054	hsa-miR-5001–3p	hsa-miR-718	hsa-miR-4793–3p	hsa-miR-4524a-5p	hsa-miR-513a-5p
hsa_circ_100219	hsa_circ_0004619	hsa-miR-135b-3p	hsa-miR-298	hsa-miR-485–3p	hsa-miR-182–5p	hsa-miR-593–5p
hsa_circ_406697	−	hsa-miR-6873–3p	hsa-miR-6833–3p	hsa-miR-6845–3p	hsa-miR-6742–5p	hsa-miR-100–3p

MRE: microRNA response element.

## DISCUSSION

CircRNAs may be generated from exonic or intronic sequences [39] and function as miRNA sponges, regulators of splicing and transcription, and modifiers of parental gene expression [39, 40]. There is evidence that circRNAs are differentially expressed in basal cell carcinoma [27], laryngeal cancer [41] and breast cancer [29, 30]. In the current two-stage epidemiological study, we constructed a profile of differentially expressed circRNAs and explored their involvement in breast cancer. Bioinformatics indicated that specific circRNAs might promote the carcinogenesis of breast cancer by sequestering miRNAs and participating in cancer-related pathways. Our study highlighted the importance of circRNAs in tumorigenesis and suggested that deregulated circRNAs may be diagnostic biomarkers of breast cancer.

In this study, we sequenced six circRNAs of interest following a microarray screening. Among them, hsa_circ_103110, hsa_circ_104689 and hsa_circ_104821 were upregulated in breast cancer tissues. Hsa_circ_103110 is encoded by the *NRIP1* (nuclear receptor interacting protein 1) gene, the protein product of which stimulates the transcriptional activity of the estrogen receptor and is critical for promoting the progression and development of breast cancer [42, 43]. Hsa_circ_104689 is spliced from *ASAP1* (ArfGAP with SH3 domain, ankyrin repeat and PH domain 1), which encodes an oncoprotein associated with colorectal cancer, laryngeal squamous cell cancer and epithelial ovarian cancer [44–46]. Hsa_circ_104821 is derived from *FAM120A* (family with sequence similarity 120A), and its encoded protein is a signaling partner that activates the FAK and PI3K pathways in colon cancer metastasis [47]. On the other hand, hsa_circ_006054, hsa_circ_100219 (also known as hsa_circ_0004619) and hsa_circ_406697 were downregulated in breast cancer tissues. Hsa_circ_006054 aligns with the gene *KIAA0355*, which encodes an uncharacterized protein that may be involved in colorectal carcinogenesis [48]. Hsa_circ_100219 is derived from *FAF1* (Fas associated factor 1), which encodes a protein that binds to FAS antigen and initiates or enhances apoptosis. FAF1 also functions as a tumor suppressor, and ectopic *FAF1* expression reduces the migration of cancer cells *in vitro* and invasion/metastasis *in vivo* [49]. Hsa_circ_100219 is matched with miR-135b [50], while hsa-miR-135b has been found to be up-regulated in cutaneous squamous cell carcinoma [51]. This circRNA had the highest diagnostic accuracy in the current study. Hsa_circ_406697 aligns with the gene *RBM22* (RNA binding motif protein 22). *RBM22* encodes an RNA binding protein which plays a role in cell division and may be involved in pre-mRNA splicing [52, 53].

GO terms provide proofs of concept for target genes that may regulate crucial biological processes during the development of human diseases. The Hippo signaling pathway has been reported to activate microprocessor which is necessary in mediating the genesis of miRNAs from the primary miRNA transcript, and link cell-density-dependent miRNA biogenesis to cancer [54]. The WNT signaling pathway underlies a wide range of human pathologies and is one of the most canonical cancer-related signaling pathways [55]. The RAP1 signaling pathway is important for both major processes of vessel formation and angiogenesis, while the RAS signaling pathway is a key determinant of the metastatic dissemination of luminal breast cancer [56, 57].

The function of circRNAs remains unclear. An intriguing possibility is that circRNAs act as microRNA sponges. Oncogenic miRNAs like hsa-miR-339-5p, hsa-miR-143-5p, hsa-miR-409-3p, hsa-miR-153-3p and hsa-miR-145-5p have been reported to be downregulated in breast cancer [31–35]. These miRNAs were matched with upregulated circRNAs in our study. Other miRNAs, like hsa-miR-298, hsa-miR-485-3p, and hsa-miR-100, which were matched with downregulated circRNAs in our study, have also been reported to be related to breast cancer [36–38].

Some limitations must be considered in the interpretation of our results. First, due to the relatively low levels of circRNA and the minimum detection thresholds of current methods, the possibility of obtaining false negatives when evaluating circRNA expression cannot be avoided. Second, the sample size was limited and the associations need to be further confirmed. The molecules associated with the present circRNAs, such as miRNAs or proteins, should be experimentally identified and characterized in the future. Third, circulating biomarkers are more acceptable than tissue biomarkers and have greater value in clinical applications. Further studies will be needed to evaluate the diagnostic value of circRNA levels in peripheral blood samples.

In summary, our study provided a profile of circRNAs in breast cancer and adjacent normal-appearing tissues. We discovered that hsa_circ_103110, hsa_circ_104689 and hsa_circ_104821 were upregulated, while hsa_circ_006054, hsa_circ_100219 and hsa_circ_406697 were downregulated in breast cancer tissues. Specific circRNAs are important promoters of carcinogenesis, as they participate in cancer-related pathways and sequester miRNAs, and thus may be useful biomarkers of breast cancer.

## MATERIALS AND METHODS

### Ethics statement

The Institutional Review Board of Nanjing Medical University (Nanjing, China) approved this study. Written informed consent was obtained from all participants included in the study.

### Study design

We designed a two-stage study. First, we used the Arraystar Human circRNA Array V2 (8×15K, Arraystar) to construct a genome-wide circRNA profile. Then, we selected candidate circRNAs for validation using qRT-PCR with a relatively large sample size.

### Patients and specimens

We recruited breast cancer patients from the Affiliated Hospital of Jiangsu University, the People's Hospital of Yixing and the First Affiliated Hospital of Suzhou University from March to May 2016. Patients were included if they were: (1) women; (2) with a pathologic diagnosis of breast cancer; (3) without previous cancer history; (4) without HIV/AIDS; (5) >18 years old; (6) having undergone mastectomy; (7) with informed consent. Breast cancer lesions and adjacent normal-appearing tissues were collected from patients who underwent surgical breast resection. The corresponding adjacent normal-appearing tissues were located >5 cm from the edge of the tumors. All patients had no history of radiotherapy or chemotherapy before specimen collection. The specimens were placed in RNA storage solutions (Shanghai Biotechnology Corporation, Shanghai, China) and stored at −80 °C in an ultra-low temperature refrigerator. Tumor stage was determined according to the Classification of Malignant Tumors Staging System (TNM) by the American Joint Committee on Cancer [58].

### RNA isolation

Total RNA was isolated with an RNeasy Mini Kit (Qiagen, Hilden, Germany) according to the manufacturer's protocol. The quality and quantity of RNA were measured with a NanoDrop ND-2000 (Thermo Fisher Scientific, Waltham, Massachusetts, United States). Additionally, RNA integrity was assessed through standard denaturing agarose gel electrophoresis.

### CircRNA microarray

The Arraystar Human Circular RNA Microarray V2 (Catalog No: AS-CR-H-V2.0, Arraystar Inc., MD, USA) was used to identify circRNAs with differential expression between breast cancer lesions and adjacent normal-appearing tissues. The array covers 13,617 human circRNAs with stringent experimental support, carefully and comprehensively collected from circRNA studies and landmark publications. Sample labeling and array hybridization were performed according to the manufacturer's protocol.

### qRT-PCR

During the validation stage, we selected 51 breast cancer samples, matched with adjacent normal-appearing tissues. cDNAs were synthesized with the Prime-Script RT reagent kit (TaKaRa, Japan) from 500 ng of total RNA. Subsequently, we performed a SYBR method-based qRT-PCR reaction in a total volume of 10 μL, including 0.2 μL/10 μM forward/reverse primers, 0.2 μL 50× ROX reference dye I, 1 μL cDNA, 5 μL 2× SYBR Premix Ex Taq II and 3.4 μL double-distilled water. The cycling program entailed initiation at 95°C for 30 sec, followed by 40 cycles of 95°C for 5 sec and a pre-selected annealing temperature for 30 sec. The best annealing temperatures were 63°C for hsa_circ_103110, hsa_circ_104689, hsa_circ_104821 and hsa_circ_100219, and 65°C for hsa_circ_006054 and hsa_circ_406697. Results were obtained from three independent wells. The relative expression of each circRNA was calculated from the ΔCt. Divergent primers were designed to amplify the circRNA-specific back-splice junctions (Table 4). We used *GAPDH* as an internal control. The primers were designed through Primer3 web (http://primer3.ut.ee/), verified through primer-BLAST (https://www.ncbi.nlm.nih.gov/tools/primer-blast/), and synthesized by Realgene (Nanjing, China). The appearance of a single peak in the melting curve of the qRT–PCR indicated the specificity of the PCR results (Supplementary Figure 4).

**Table 4:** Primers designed for RT-PCR

CircRNA	Forward (5’-3’)	Reverse (5’-3’)
hsa_circ_103110	CCGGATGACATCAGAGCTACT	ACACTTCCGTCTGTCTCCAA
hsa_circ_104689	TGGCAGTGAAAAGAAGGGGT	TGAAAGAAATGTGGCATGTGAGA
hsa_circ_104821	CCACCACATTACTTAGGTTGCA	CGTTCCGGCTCAGTTTTAGG
hsa_circ_006054	TCCTGTGACTGAAGTGCTGA	GTCTAGATGTCGCCAGTCCA
hsa_circ_100219	TGCTACAGACGACTCAGAGA	AGATGATGAAGGTGGTGGCA
hsa_circ_406697	GAGACAGATTTAAGGCCTGCC	GGTAGATGTGGCTTTCCCCA

### Bioinformatics and data analysis

We performed GO analysis to annotate genes meaningfully in terms of their biological processes, cellular components and molecular functions. The -log10 (P-value) yields an enrichment score representing the significance of GO term enrichment among differentially expressed genes. KEGG analysis was performed to determine the involvement of target genes in different biological pathways. Here, the -log10 (P-value) yields an enrichment score indicating the significance of pathway correlations. To further elucidate the correlations between circRNAs and miRNAs, we predicted circRNA/miRNA interactions using miRNA target prediction software from Arraystar, which refers to TargetScan (http://www.targetscan.org/) and miRanda (http://www.microrna.org/).

### Statistical analysis

The fold-change of each circRNA was computed from the profile difference between the cancer and control groups, and the significance was analyzed with a t-test. A receiver operating characteristic curve was plotted, and the AUC, sensitivity and specificity were calculated to assess the ability of circRNAs to differentiate between breast cancer and adjacent normal-appearing tissues. Statistical analyses were performed with R software version 3.3.1 (https://www.r-project.org/) and GraphPad Prism 5 (GraphPad Software, La Jolla, CA).

## SUPPLEMENTARY MATERIALS FIGURES AND TABLES




