# Supplementary material for: Identification of circular RNAs as a promising new class of diagnostic biomarkers for human breast cancer
Source: Oncotarget. 2017 Apr 21;8(27):44096–107. doi: 10.18632/oncotarget.17307 (PMC5546465; doi:10.18632/oncotarget.17307)
Supplement: Supplementary file 1 [file oncotarget-08-44096-s001.pdf]

Identification of circular RNAs as a promising new class of diagnostic biomarkers for human breast cancer

SUPPLEMENTARY MATERIALS

SUPPLEMENTARY TABLES AND FIGURES

Supplementary Table 1: Detailed information about the four patients included in the microarray analysis

No	Ages	Ethnic	Menopausal	TNM	ER	PR	HER2	Histological type
1	46	Chinese Han	pre-	T2N0M0	positive	positive	negative	invasive ductal carcinoma
2	62	Chinese Han	post-	T2N0M0	positive	positive	positive	invasive ductal carcinoma
3	41	Chinese Han	pre-	T2N2M0	positive	negative	negative	invasive ductal carcinoma
4	74	Chinese Han	post-	T1N0M0	negative	negative	positive	invasive ductal carcinoma

Supplementary Table 2: The circRNA levels (ΔCt) and clinical factors of patients with breast cancer

See Supplementary File 1

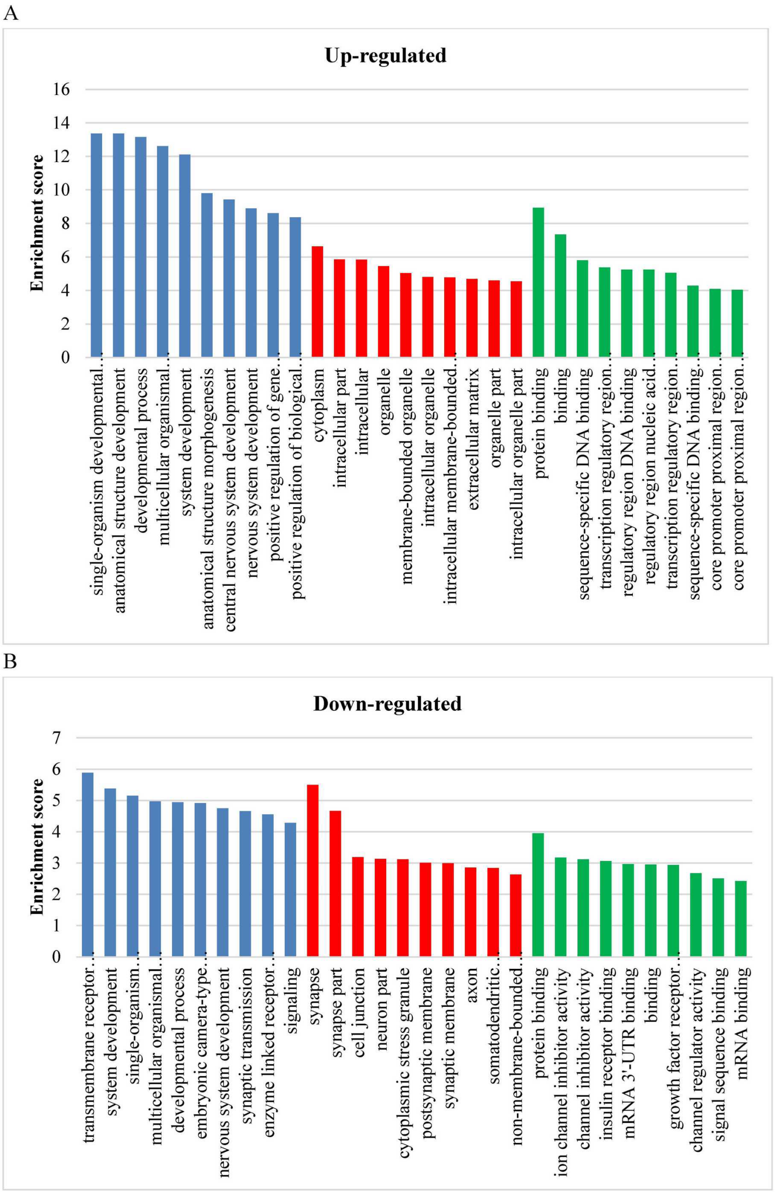

Supplementary Figure 1: GO analysis of the biological functions of circRNA target genes. (A) GO annotation corresponding to the target genes of upregulated circRNAs. **(B)** GO annotation corresponding to the target genes of downregulated circRNAs.

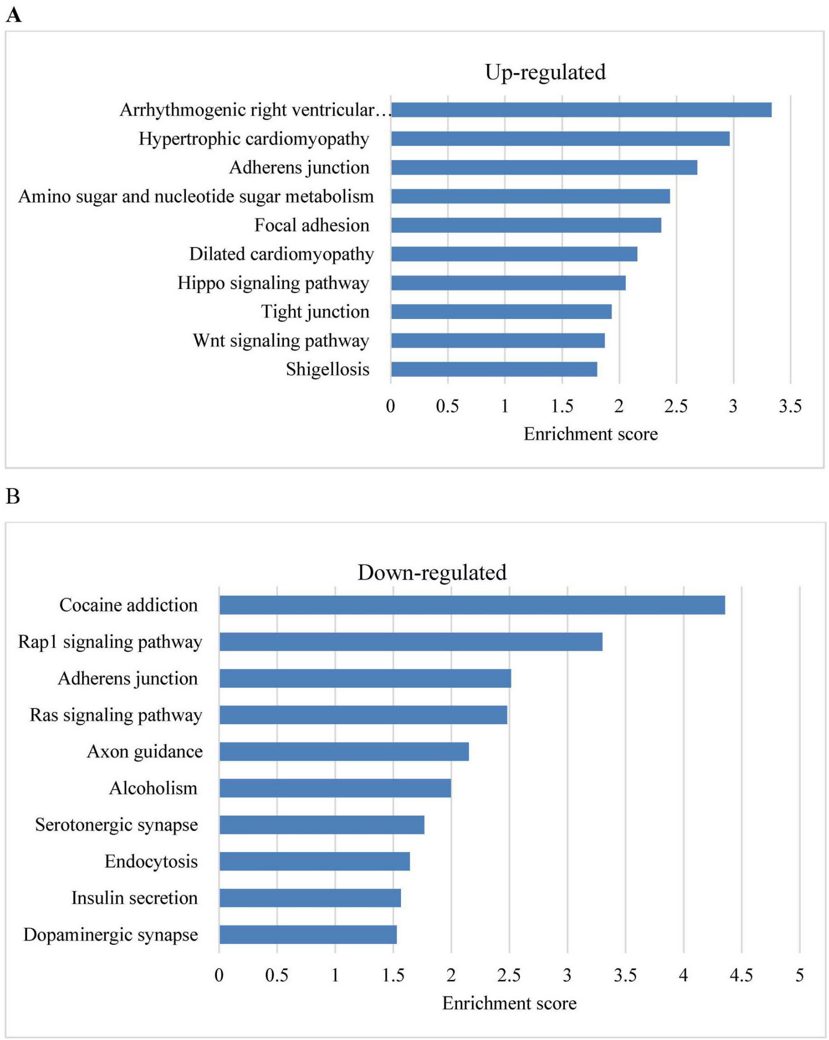

Supplementary Figure 2: KEGG pathway analysis. (A) Pathways corresponding to upregulated circRNAs. **(B)** Pathways corresponding to downregulated circRNAs.

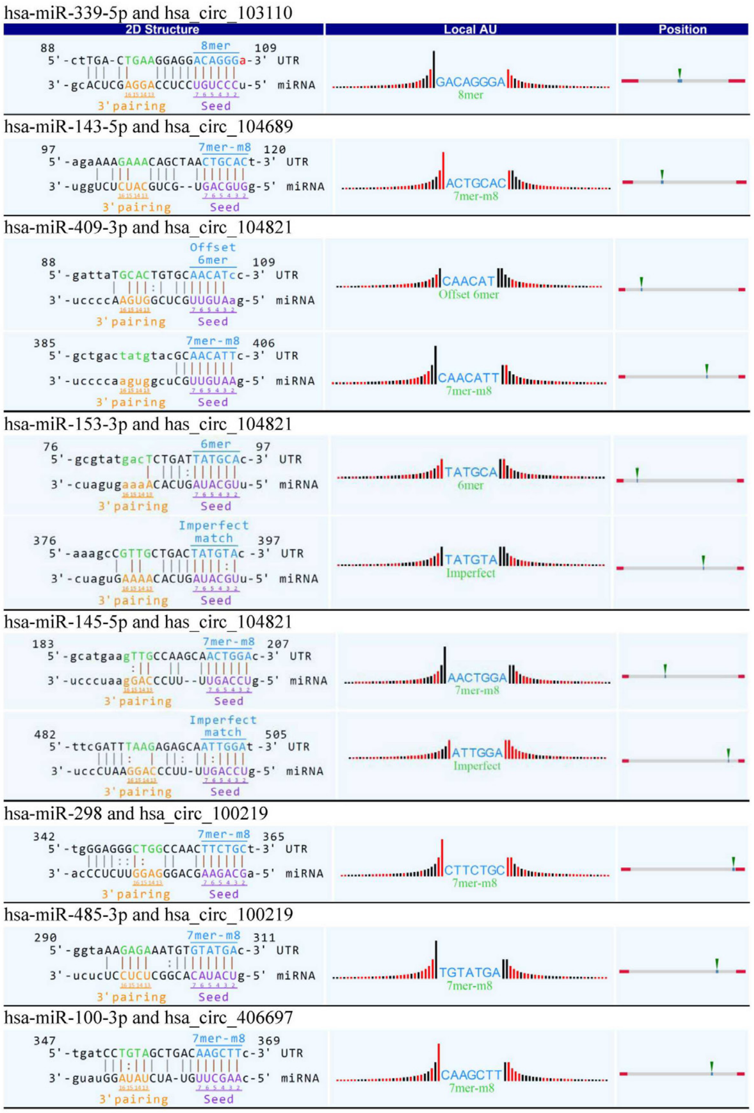

Supplementary Figure 3: Detailed annotation of circRNA/miRNA interactions. The 2D structure displays the MRE sequence, the target miRNA seed type (7mer-m8, 8mer, 6mer, offset 6mer, imperfect) and the pairing of target miRNA nucleotides 13-16. The precise base positions are shown in the alignments in the upper left and right corners. The "local AU" displays the 30 nucleotides upstream and downstream of the seed sequence. Black bars stand for G/C and low accessibility. Red bars stand for A/U and high accessibility of the seed. The height of the bar represents the extent of accessibility. The position column displays the most likely relative MRE position on the linearized circRNA sequence.

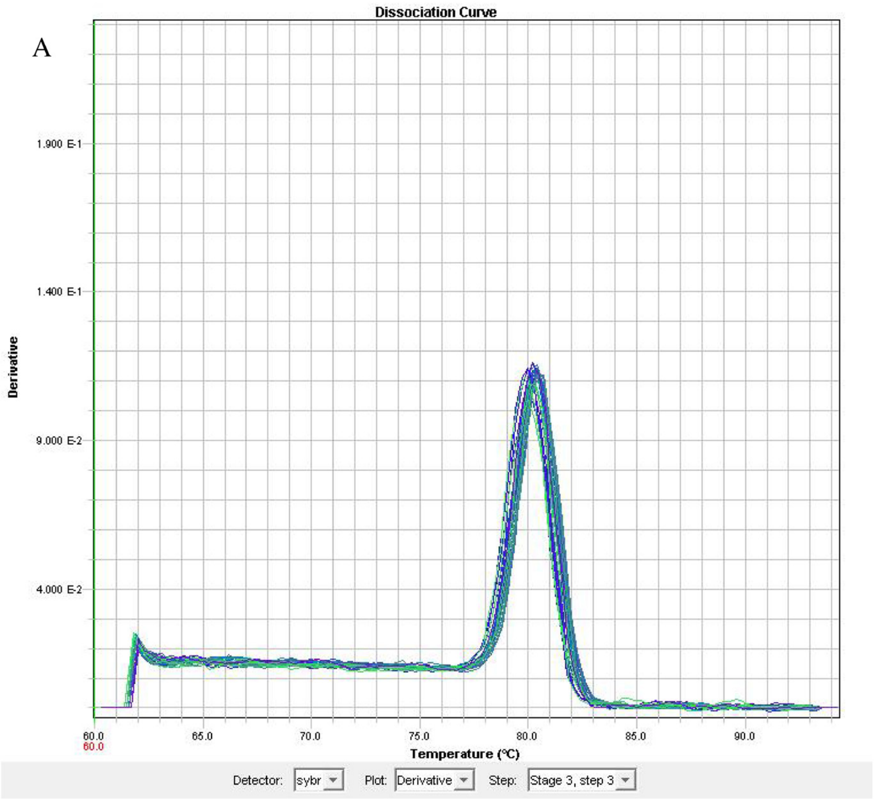
A
Dissociation Curve
Derivative
1.900 E-1
1.400 E-1
9.000 E-2
4.000 E-2
60.0
65.0
70.0
75.0
80.0
85.0
90.0
Temperature (°C)
Detector: sybr
Plot: Derivative
Step: Stage 3, step 3

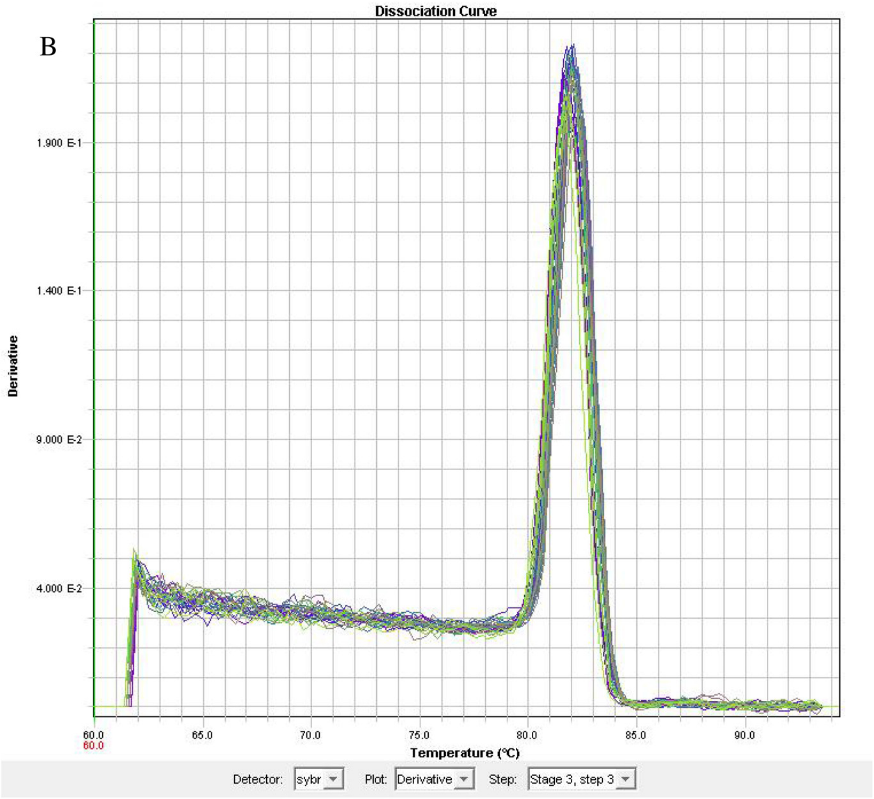
B
Dissociation Curve
Derivative
1.900 E-1
1.400 E-1
9.000 E-2
4.000 E-2
60.0
65.0
70.0
75.0
80.0
85.0
90.0
Temperature (°C)
Detector: sybr
Plot: Derivative
Step: Stage 3, step 3

(*Continued*)

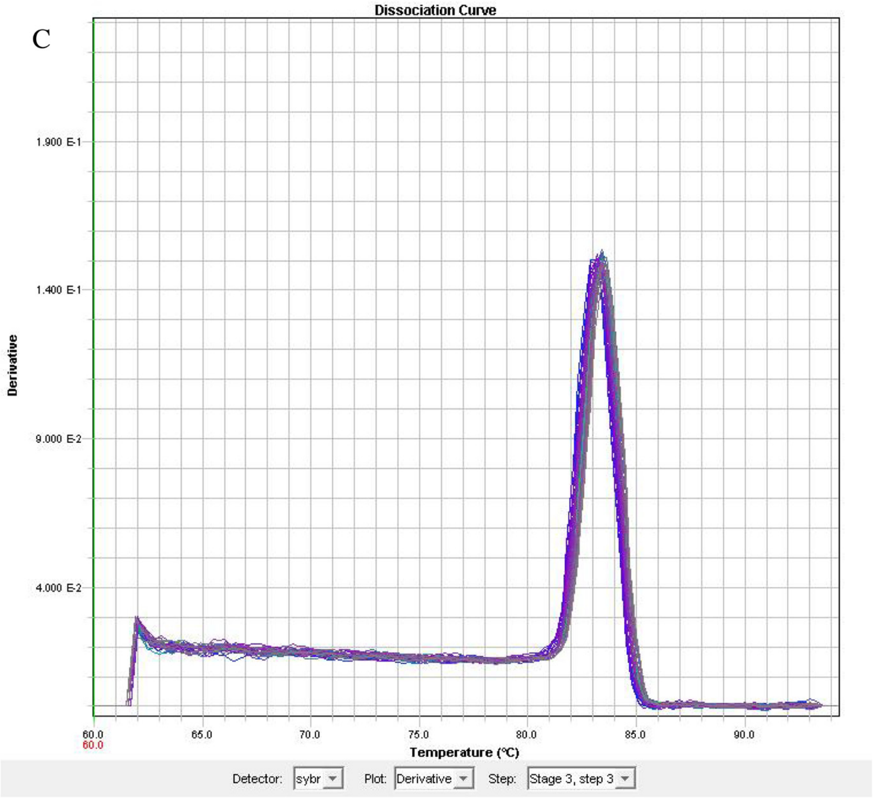
C
Dissociation Curve
1.900 E-1
1.400 E-1
9.000 E-2
4.000 E-2
Derivative
60.0
65.0
70.0
75.0
80.0
85.0
90.0
Temperature (°C)
Detector: sybr
Plot: Derivative
Step: Stage 3, step 3

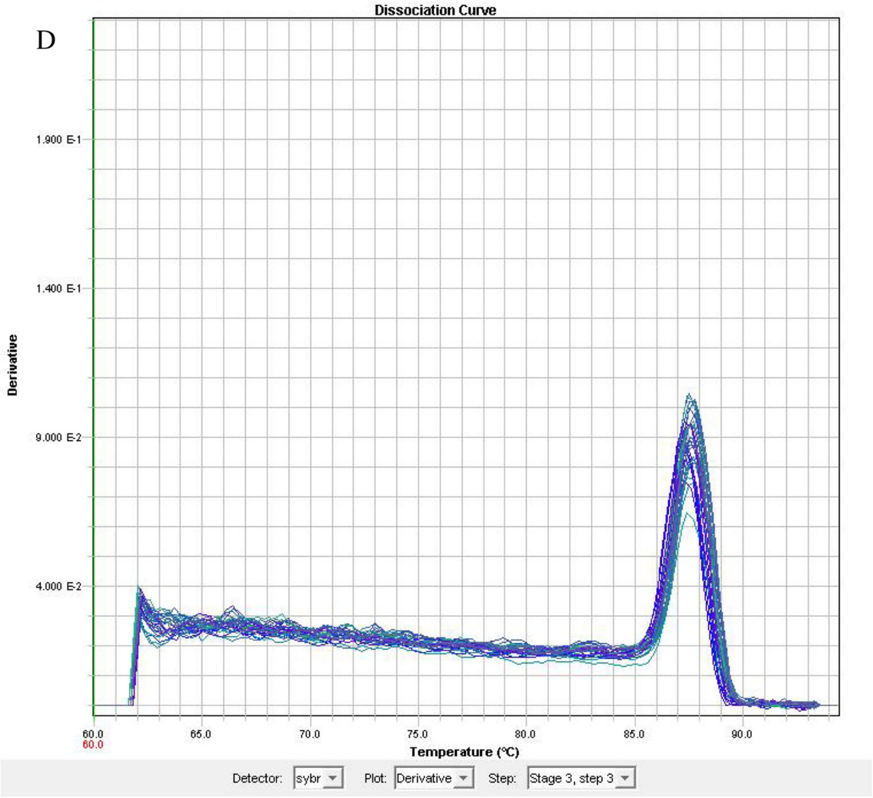
D
Dissociation Curve
1.900 E-1
1.400 E-1
9.000 E-2
4.000 E-2
Derivative
60.0
65.0
70.0
75.0
80.0
85.0
90.0
Temperature (°C)
Detector: sybr
Plot: Derivative
Step: Stage 3, step 3

(*Continued*)

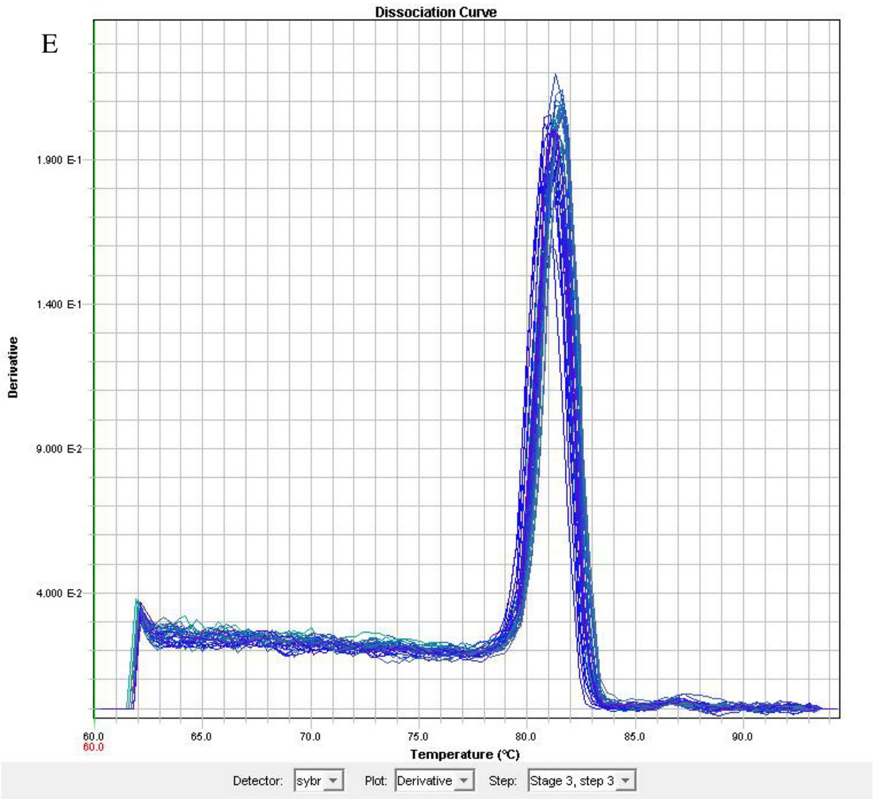
E
Dissociation Curve
1.900 E-1
1.400 E-1
9.000 E-2
4.000 E-2
Derivative
60.0
60.0
65.0
70.0
75.0
80.0
85.0
90.0
Temperature (°C)
Detector: sybr
Plot: Derivative
Step: Stage 3, step 3

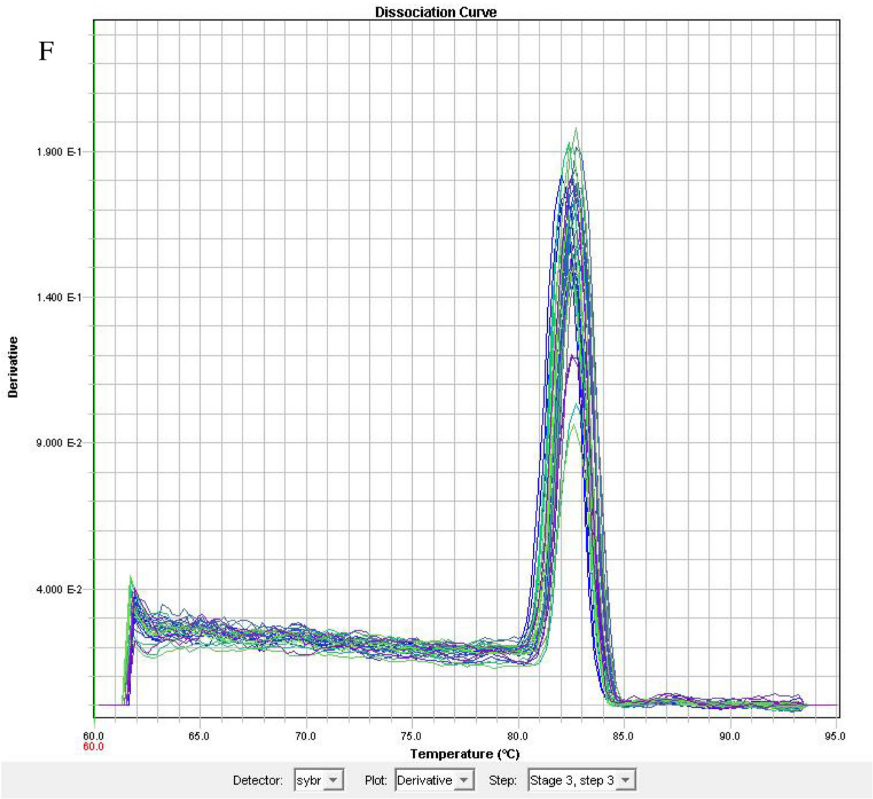
F
Dissociation Curve
1.900 E-1
1.400 E-1
9.000 E-2
4.000 E-2
Derivative
60.0
60.0
65.0
70.0
75.0
80.0
85.0
90.0
95.0
Temperature (°C)
Detector: sybr
Plot: Derivative
Step: Stage 3, step 3

(*Continued*)

G

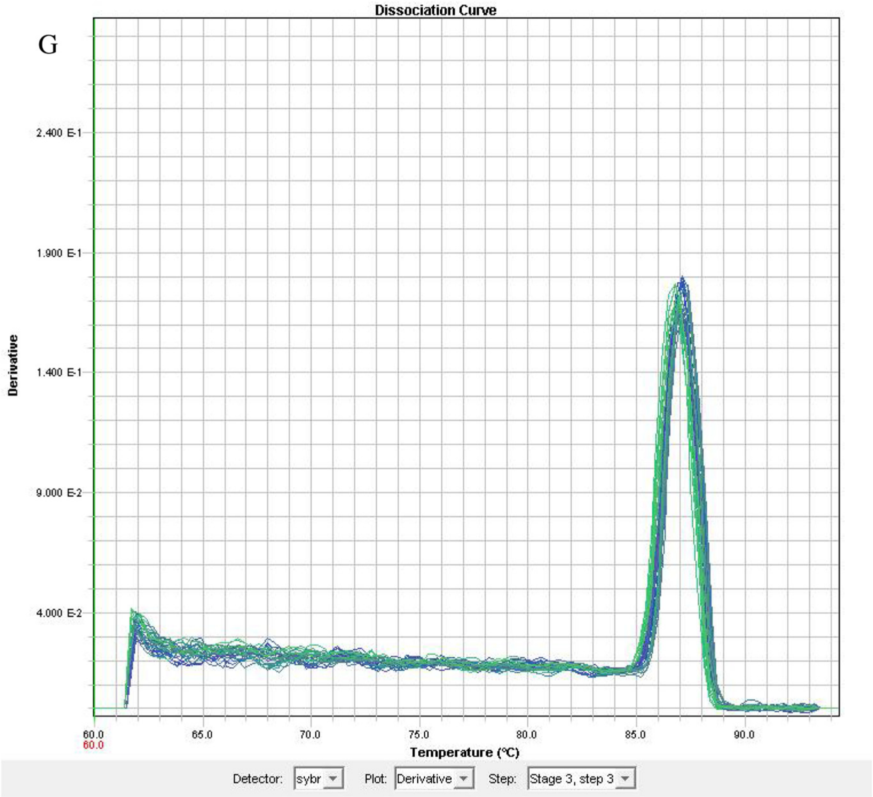

Supplementary Figure 4: Melting curves of differentially expressed circRNAs. **(A)** hsa_circ_103110; **(B)** hsa_circ_104689; **(C)** hsa_circ_104821; **(D)** hsa_circ_006054; **(E)** hsa_circ_100219; **(F)** hsa_circ_406697; **(G)** *GAPDH*.
