# Supplementary material for: Identification of circular RNAs as a promising new class of diagnostic biomarkers for human breast cancer
Source: Oncotarget. 2017 Apr 21;8(27):44096–107. doi: 10.18632/oncotarget.17307 (PMC5546465; doi:10.18632/oncotarget.17307)
Supplement: Supplementary file 2 [file oncotarget-08-44096-s002.docx]

Supplementary Table 2. The relationship of circRNAs expression levels (ΔCt) in cancer tissues with clinicopathological factors of patients with breast cancer

| Characteritics | Hsa_circ_103110 | | Hsa_circ_104689 | | Hsa_circ_104821 | | Hsa_circ_006054 | | Hsa_circ_100219 | | Hsa_circ_406697 | |
| --- | --- | --- | --- | --- | --- | --- | --- | --- | --- | --- | --- | --- |
|  | mean | *P* | mean | *P* | mean | *P* | mean | *P* | mean | *P* | mean | *P* |
| Age |  |  |  |  |  |  |  |  |  |  |  |  |
| ≤60 | 8.79 ± 2.16 | 0.915 | 8.09 ± 2.27 | 0.249 | 6.15 ± 1.58 | 0.686 | 15.92 ± 1.79 | 0.221 | 10.16 ± 1.57 | 0.848 | 15.04 ± 2.43 | 0.493 |
| >60 | 8.85 ± 1.33 |  | 7.43 ± 1.08 |  | 5.96 ± 1.54 |  | 15.27 ± 1.83 |  | 10.07 ± 1.64 |  | 14.58 ± 2.02 |  |
| ER |  |  |  |  |  |  |  |  |  |  |  |  |
| positive | 9.02 ± 1.33 | 0.429 | 8.17 ± 2.15 | 0.238 | 6.21 ± 1.42 | 0.554 | 15.41 ± 1.91 | 0.333 | 10.07 ± 1.48 | 0.818 | 14.44 ± 2.72 | 0.200 |
| negative | 8.58 ± 2.31 |  | 7.51 ± 1.63 |  | 5.94 ± 1.69 |  | 15.91 ± 1.72 |  | 10.18 ± 1.70 |  | 15.27 ± 1.64 |  |
| PR |  |  |  |  |  |  |  |  |  |  |  |  |
| positive | 8.53 ± 1.28 | 0.339 | 8.61 ± 2.42 | **0.038^*^** | 6.72 ± 1.45 | **0.039^*^** | 15.04 ± 1.84 | 0.070 | 10.02 ± 1.52 | 0.721 | 13.70 ± 2.60 | **0.005^*^** |
| negative | 9.00 ± 2.13 |  | 7.42 ± 1.46 |  | 5.75 ± 1.52 |  | 16.01 ± 1.73 |  | 10.19 ± 1.64 |  | 15.51 ± 1.78 |  |
| HER2 |  |  |  |  |  |  |  |  |  |  |  |  |
| positive | 8.63 ± 1.85 | 0.418 | 8.09 ± 2.06 | 0.237 | 6.30 ± 1.47 | 0.214 | 15.45 ± 1.71 | 0.330 | 9.82 ± 1.46 | 0.084 | 14.76 ± 2.34 | 0.727 |
| negative | 9.06 ± 1.86 |  | 7.42 ± 1.63 |  | 5.72 ± 1.65 |  | 15.97 ± 1.96 |  | 10.62 ± 1.68 |  | 14.99 ± 2.20 |  |
| Tumor size |  |  |  |  |  |  |  |  |  |  |  |  |
| T1 | 8.87 ± 1.40 | 0.387 | 8.00 ± 1.44 | 0.300 | 6.25 ± 1.66 | 0.848 | 15.30 ± 2.12 | 0.458 | 10.01 ± 1.64 | 0.798 | 14.46 ± 2.80 | 0.563 |
| T2 | 8.98 ± 1.93 |  | 7.97 ± 2.15 |  | 6.03 ± 1.57 |  | 15.93 ± 1.69 |  | 10.25 ± 1.63 |  | 15.14 ± 2.12 |  |
| T3 | 7.32 ± 2.63 |  | 6.57 ± 1.48 |  | 5.82 ± 1.99 |  | 15.21 ± 1.46 |  | 9.80 ± 1.35 |  | 14.34 ± 1.11 |  |
| Lymphatic metastasis |  |  |  |  |  |  |  |  |  |  |  |  |
| N0 | 8.64 ± 1.79 | 0.341 | 7.79 ± 2.12 | **0.031^*^** | 6.32 ± 1.40 | 0.295 | 15.67 ± 1.72 | 0.783 | 10.46 ± 1.40 | 0.209 | 14.92 ± 2.21 | 0.832 |
| N1 | 8.64 ± 1.66 |  | 8.57 ± 1.62 |  | 6.14 ± 1.84 |  | 15.47 ± 2.09 |  | 9.56 ± 1.69 |  | 14.61 ± 2.75 |  |
| N2 | 9.70 ± 2.32 |  | 6.53 ± 1.23 |  | 5.35 ± 1.76 |  | 16.00 ± 1.66 |  | 10.25 ± 1.76 |  | 15.15 ± 1.41 |  |

ER: [estrogen](javascript:void(0);) receptor; PR: progestin receptor; HER2: human epidermal growth factor receptor-2
